# Anti‐thrombotic therapy in patients with cancer at the end of life: A cohort study using population‐linked routinely collected data

**DOI:** 10.1111/bjh.70032

**Published:** 2025-09-01

**Authors:** Sarah J. Aldridge, Ashley Akbari, Adrian Edwards, Kate J. Lifford, Denise Abbel, Suzanne Cannegieter, Jamilla Goedegebuur, Eva K. Kempers, Mette Søgaard, Chantal Visser, Geert‐Jan Geersing, Marieke J. H. Kruip, Anne Gulbech Ording, Carline van den Dries, Eric C. T. Geijteman, Frederikus A. Klok, Isabelle Mahé, Simon P. Mooijaart, Sebastian Szmit, Simon Noble

**Affiliations:** ^1^ Population Data Science, Faculty of Medicine Health and Life Science Swansea University Swansea UK; ^2^ PRIME Centre Wales, Division of Population Medicine, School of Medicine Cardiff University Cardiff UK; ^3^ Department of Medicine, Section of Gerontology and Geriatrics Leiden University Medical Center, Leiden University Leiden The Netherlands; ^4^ Department of Medicine, Section of Thrombosis and Hemostasis Leiden University Medical Center, Leiden University Leiden The Netherlands; ^5^ Centre for Medicine for Older People Leiden University Medical Center, Leiden University Leiden The Netherlands; ^6^ Department of Clinical Epidemiology Leiden University Medical Center, Leiden University Leiden The Netherlands; ^7^ Department of Hematology Erasmus University Medical Centre Rotterdam Rotterdam The Netherlands; ^8^ Danish Center for Health Services Research, Department of Clinical Medicine Aalborg University, Aalborg University Hospital Gistrup Denmark; ^9^ Center for General Practice Aalborg University Aalborg Denmark; ^10^ Department of General Practice & Nursing Science, Julius Center for Health Sciences and Primary Care University Medical Center Utrecht, Utrecht University Utrecht The Netherlands; ^11^ Department of Medical Oncology Erasmus Medical Center Rotterdam The Netherlands; ^12^ Assistance Publique–Hôpitaux de Paris (AP‐HP), Hôpital Louis Mourier, Service de Médecine Interne Colombes France; ^13^ Université Paris Cité Paris France; ^14^ Centre for Postgraduate Medical Education, Department of Cardio‐Oncology Warsaw Poland

**Keywords:** anti‐coagulants, anti‐platelets, anti‐thrombotic therapy, cohort study, end of life cancer care, palliative care

## Abstract

Anti‐thrombotic therapy (ATT) in cancer patients approaching the end of life presents significant clinical challenges, balancing thrombotic and bleeding risks. This study analysed ATT prescribing patterns and associated outcomes in patients diagnosed with poor prognosis cancer, defined as cancer diagnoses associated with a 1‐year life expectancy, using the Welsh national Secure Anonymised Information Linkage Databank. Retrospective cohort study of adults in Wales diagnosed with poor prognosis cancer between 2013 and 2021, following up patients from cancer diagnosis until death, end of follow‐up or study end (31 December 2021). Outcomes included ATT discontinuation, bleeding and thromboembolic events in secondary care. We identified a cohort of 25 783 adults with a median survival of 145 days. Of these, 32% were receiving ATT at diagnosis, with 77% continuing until death. One‐year cumulative incidence of ATT discontinuation was 19% (95% CI: 18%–20%). The 1‐year cumulative incidence of bleeding was 3.2% (95% CI: 3.0%–3.4%) and of thromboembolic events was 5.3% (95% CI: 5.0%–5.6%). ATT was prevalent at cancer diagnosis and discontinuation before death was uncommon. The management of ATT is complex in patients with advanced cancer and there is a need for clearer guidance on appropriate discontinuation strategies as well as when to continue these medicines.

## Graphical abstract

Anti‐thrombotic therapy (ATT) prescribing patterns and outcomes in patients with poor prognosis cancer in Wales (2013–2021). This retrospective cohort study of 25 783 adults with cancer diagnoses associated with ≤1‐year life expectancy showed that 32% were receiving ATT at diagnosis, with 77% continuing until death. One‐year cumulative incidence rates were 19% for ATT discontinuation, 3.2% for bleeding events and 5.3% for thromboembolic events, highlighting the clinical complexity of balancing thrombotic and bleeding risks in this population.

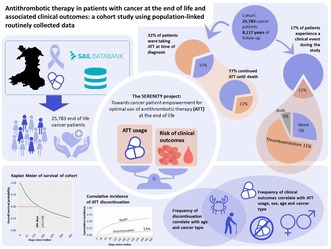

## INTRODUCTION

Approximately 30%–50% of people with cancer are prescribed anti‐coagulants and anti‐platelet agents, collectively termed anti‐thrombotic therapy (ATT), but little is known about the balance of their benefits and harms in the context of malignancy.^1^, ^2^, ^3^ Most patients are already receiving ATT at the time of cancer diagnosis for arterial thromboembolism (ATE) such as cardiovascular, cerebrovascular and peripheral vascular disease or as stroke prevention for atrial fibrillation or mechanical heart valves. Malignancy confers an additional thrombotic risk not only for ATE but also, more commonly, for venous thromboembolism (VTE), which affects up to 20% of cancer patients.^4^, ^5^, ^6^ The prothrombotic state is multifactorial and includes procoagulant expression by the tumour itself and extrinsic factors such as systemic anti‐cancer therapies (SACT).^7^ In addition to an increased risk of cancer‐associated thrombosis (CAT), patients are also at greater risk of bleeding; in part due to the highly vascular nature of some tumours, altered haemostasis and thrombolysis and from receiving cytopenic SACT regimes,^1^ this is further exacerbated by the use of ATT.^8^

The management of ATT is particularly challenging in cancer patients with advanced disease since both thrombotic and bleeding rates increase with disease progression.^9^ Over 28% of hospice patients have asymptomatic deep vein thrombosis (DVT) and pulmonary emboli are seen in half of cancer patients at post‐mortem.^10^, ^11^ Furthermore, 7.0%–9.8% of patients at the end of life experience clinically relevant non‐major bleeding such as haemoptysis, epistaxis and haematemesis, and this is strongly associated with the use of both anti‐platelet agents and anti‐coagulants.^12^, ^13^ Consequently, ATT management near the end of life requires careful balancing of the risks of bleeding and thrombosis, while considering the patient's personal preferences, values and goals of care.^14^, ^15^

Approximately 5%–15% of cancer patients at the end of life are receiving anti‐coagulants and 25%–35% anti‐platelets respectively.^1^, ^2^ While cancer‐specific clinical guidelines are clear in advising indications for the commencement of ATT, there are few data regarding when they should be stopped. Current best practice recommends indefinite therapy as long as the thrombotic risk persists, and the anticipated bleeding risk remains acceptable.^16^, ^17^ However, the data suggest that most cancer patients receiving ATT remain treated until death, and this is associated with a higher rate of bleeding, distress and complex bereavement sequelae.^12^, ^13^, ^18^

Steps to optimise ATT in cancer patients nearing the end of life are currently underway. The SERENITY project is a pan‐European collaboration to develop and evaluate a decision support tool to facilitate discussions regarding whether to continue or stop ATT as death approaches in cancer patients.^19^ These include the analysis of various European databases to further understand the use of ATT and to analyse the associated risks of major bleeding, VTE and ATE according to ATT exposure among patients with cancer in the last year of life.^18^, ^20^ We report the outcomes from the UK arm of the project using the Secure Anonymised Information Linkage (SAIL) Databank, the national trusted research environment (TRE) for Wales, containing population‐scale, anonymised individual‐level linked data sources.

## MATERIALS AND METHODS

We constructed a cohort representing adults living in Wales diagnosed with poor prognosis cancers between 1 January 2013 and 31 December 2021, as defined by a life expectancy of less than 1 year according to their primary diagnosis and stage (Section S1.1). Follow‐up ran from diagnosis (index date) until death, migration or study end (31 December 2021), whichever occurred first.

### Data sources

Data were accessed through the SAIL Databank, which integrates linked, pseudonymised data from GP records, hospital admissions, emergency departments and administrative sources.^19^, ^21^ Among these SAIL data are the Welsh Cancer Intelligence and Surveillance Unit (WCSU) and the Cancer Network Information System Cymru (CNIS). WCSU is responsible for collecting, managing and analysing cancer‐related data in Wales and holds data from the National Cancer Registry. It plays a primary role in recording, storing and reporting all cancer incidences for the Welsh population^20^ (Section S2.1 details all data sources used). Crucially, this dataset is subject to a series of validation checks, which result in a delay in coverage. Currently, SAIL has access to WCSU data until 31 December 2020, and CNIS data until the end of the study period. While WCSU is considered the gold standard for capturing cancer patients in Wales, it is limited to the first cancer diagnosis (one row per tumour) and is unable to capture disease progression. In contrast, CNIS records subsequent treatments and diagnoses, providing a more longitudinal dataset that can track disease progression.

### Study population

Using publicly available Welsh Government statistics (accessed from https://www.gov.wales/cancer‐survival‐wales‐2002‐2020), we defined poor prognosis cancers as those associated with ≤1‐year survival (Section S1.1). Using International Classification of Diseases (ICD‐10) codes and staging information from WCSU and CNIS, we selected a cohort of poor prognosis cancer patients in Wales. If multiple relevant diagnoses were present, only the first was used. However, if multiple diagnoses were recorded on the same *day*, all were considered.

Patients were linked to primary care and administrative data using a unique identifier to obtain demographics, residency and death data (see Section S2.1).

### Inclusion criteria

Patients were included if they had a valid linkage field, week of birth and sex, were aged between 18 and 105 years, had continuous coverage, a relevant cancer diagnosis recorded before death and during the study period (January 2013 to December 2021) and were registered with a SAIL GP at the end of their coverage period (Section S2.2).

### Exposures and outcomes

To assess ATT exposure, we selected four treatment types for analysis: Direct‐acting oral anti‐coagulants (DOAC), Vitamin K antagonists (VKA), low molecular weight heparins (LMWH) and platelet inhibitors (PI). We performed a keyword search for these ATT types on the NHS Read code version 2 code list. These Read codes were clinician‐reviewed (SN & AE) and classified as either a prescription or discontinuation event (Table S1.3). These codes were used to identify relevant prescription events from primary care data.

An initial observational study of 30 000 ATT users in SAIL found a median prescription interval of 28 days, with 95% of all prescription intervals captured within 58 days (Section S2.3). Based on this, we chose a 2‐month (62 days) cut‐off following a patient's final ATT prescription to define ATT exposure.

ATT exposure at index was defined as having at least one prescription within the 62 days prior to the index date. ATT discontinuation was characterised by the final prescription present in the patient's record. For individuals whose final ATT record was a prescription event, 62 days were added to this date to estimate the discontinuation date. If the patient died or follow‐up ended during this time, this was not considered discontinuation. For individuals whose final prescription record corresponded to a discontinuation event, this date was used as the discontinuation date.

Clinical outcomes included thromboembolic events (VTE, ATE, myocardial infarction [MI]) and clinically relevant bleeding events (henceforth referred to as bleeding events) identified via ICD‐10 codes from secondary care records (see Sections S1.3–S1.5). Patients with atrial fibrillation or prior thromboembolic events were flagged as having indicators for ATT. While there were insufficient data to grade bleeding using definitions agreed by the Scientific and Standardization Committee (SSC) of the International Society for Thrombosis and Haemostasis (ISTH), since the events were sufficient to warrant recording in secondary care, we considered they met the definition of clinically relevant non‐major bleeding as a minimum.^22^, ^23^

### Statistical approach

Summary statistics were performed to gain a better understanding of the data and to provide insights into the relationship between ATT exposure at index, clinical outcomes and cancer types.

Kaplan–Meier curves assessed time to death by ATT use, age and cancer type. One‐year cumulative incidences of ATT discontinuation, bleeding and thromboembolism were estimated using Fine and Gray models, accounting for death as a competing risk. Raw data were collected using Eclipse SDK (V4.15). Organisation, refinement and analysis were performed using R V4.1.3 making use of the survival (v3.5‐5) and cmprsk (v2.2‐11) packages for survival analysis.^24^, ^25^, ^26^

## RESULTS

### Cohort characteristics

We identified 25 783 patients diagnosed with a poor prognosis cancer between January 2013 and December 2021. Median age was 73 years (interquartile range [IQR] = 15 years) with 57% male (Table 1). Most had gastrointestinal (46%) or respiratory cancers (39%). The cohort provided 8117 person‐years (PY) of follow‐up. Median follow‐up was 140 days (IQR = 47–367) with a median survival of 145 (95% CI: 142–148) days (Table S3.1). Survival was longer in ATT‐unexposed patients at diagnosis (160 vs. 117 days, 95% CI: 155–164 vs. 112–122). Survival varied considerably with cancer type and stage from 59 (95% CI: 51–65) days in stage IV secondary and unspecified malignant neoplasms to 353 (95% CI: 313–424) days in stage III urogenital cancers.

**TABLE 1:** Summary counts of the investigated cohorts within the Welsh cohort—all counts have been rounded to the closest 10.

Characteristics	No ATT at index	ATT at index	Total
Total	17 520 (68%)	8260 (32%)	25 780
Median age	71 years (IQR = 15 years)	76 years (IQR = 13 years)	73 IQR = (15 years)
Age			
<60 years	3160 (18%)	350 (4.2%)	3510 (13.6%)
60–79 years	10 550 (60.2%)	4820 (58.4%)	15 370 (59.6%)
≥80 years	3810 (21.7%)	3090 (37.4%)	6900 (26.8%)
Sex			
Female	8170 (46.6%)	2950 (35.7%)	11 120 (43.1%)
Male	9350 (53.4%)	5310 (64.3%)	14 660 (56.9%)
Year of inclusion			
2013	2190 (12.5%)	1000 (12.1%)	3190 (12.4%)
2014	2230 (12.7%)	1090 (13.2%)	3320 (12.9%)
2015	2160 (12.3%)	1060 (12.8%)	3220 (12.5%)
2016	2280 (13%)	1070 (13%)	3350 (13%)
2017	2210 (12.6%)	1060 (12.8%)	3270 (12.7%)
2018	2340 (13.4%)	1130 (13.7%)	3470 (13.5%)
2019	2200 (12.6%)	1080 (13.1%)	3280 (12.7%)
2020	1030 (5.9%)	390 (4.7%)	1420 (5.5%)
2021	870 (5%)	380 (4.6%)	1250 (4.9%)
Primary cancer type			
Gastrointestinal	8390 (47.9%)	3470 (42%)	11 860 (46%)
Gynaecological	520 (3%)	140 (1.7%)	660 (2.6%)
Carcinoma of unknown primary	610 (3.5%)	300 (3.6%)	910 (3.5%)
Melanoma and other skin cancers	120 (0.7%)	60 (0.7%)	180 (0.7%)
Mesothelial and soft tissue	260 (1.5%)	120 (1.5%)	380 (1.5%)
Respiratory and intrathoracic organs	6490 (37%)	3680 (44.5%)	10 170 (39.4%)
Thyroid and other endocrine glands	50 (0.3%)	20 (0.2%)	70 (0.3%)
Urogenital	1080 (6.2%)	480 (5.8%)	1560 (6%)
ATT relevant health outcome prior to index			
ATT indicator 6 months prior	12 410 (33%)	3750 (29.6%)	16 160 (32.2%)
ATT indicator 1 year prior	12 470 (33.2%)	3970 (31.3%)	16 440 (32.7%)
ATT indicator 5 years prior	12 680 (33.8%)	4960 (39.1%)	17 640 (35.1%)

Abbreviation: ATT, anti‐thrombotic therapy.

At diagnosis, 32% (8260) were taking ATT, with this proportion remaining stable over time. Among these, 77% (6380) continued ATT until the end of follow‐up (Table 2). Of the 17 520 unexposed to ATT at index, 13.6% (2330) initiated ATT during follow‐up.

**TABLE 2:** Counts for outcomes following cancer diagnosis date, stratified by ATT exposure at index.

Outcome	Total	ATT at index	No ATT at index
Died before end of follow‐up	24 320 (94%)		
Discontinued ATT before end of follow‐up	2470 (10%)	1880 (23%)	590 (3%)
Continued ATT until end of follow‐up	8110 (31%)	6380 (77%)	1730 (10%)
Started ATT after index (patients who were unexposed to ATT at cancer diagnosis only)	2320 (9%)	NA	2320 (13%)
Bleeding event within 1 year of index	820 (3%)	280 (3%)	540 (3%)
Thromboembolism within 1 year of index	1350 (5%)	390 (5%)	960 (5%)
Both a major bleed and thromboembolism within 1 year of index	70 (0.3%)	20 (0.2%)	50 (0.3%)

Abbreviation: ATT, anti‐thrombotic therapy.

ATT indicators were present in 35% (17640) of patients in the prior 5 years, 33% (16440) in 1 year and 33% (16160) in 6 months. Prevalence was similar in ATT‐exposed and unexposed groups across all time windows (Table 1).

### Discontinuation

**FIGURE 1:**
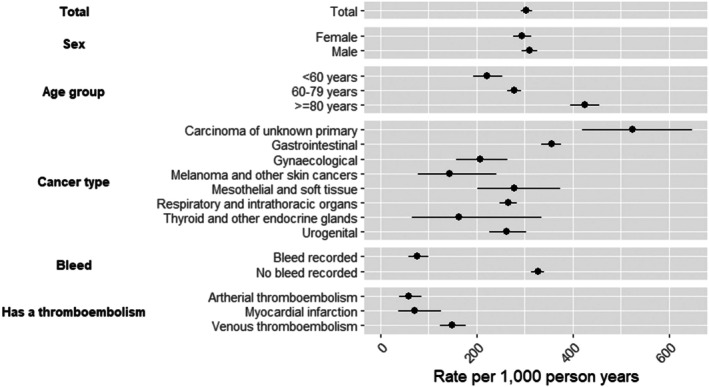
Rates of ATT discontinuation per 1000 person‐years with 95% CI. ATT, anti‐thrombotic therapy.

Among patients exposed to ATT at index, the discontinuation rate was 304/1000 PY (95% CI: 292–316). Discontinuation was more common in older males, varying substantially by cancer type. Highest rates occurred in metastatic cancers of unknown primary (525/1000 PY, 95% CI: 421–648), while thyroid and other endocrine cancers (163/1000 PY, 95% CI: 65–335) and skin cancers (144/1000 PY, 95% CI: 79–241) had the lowest (Figure 1).

**FIGURE 2:**
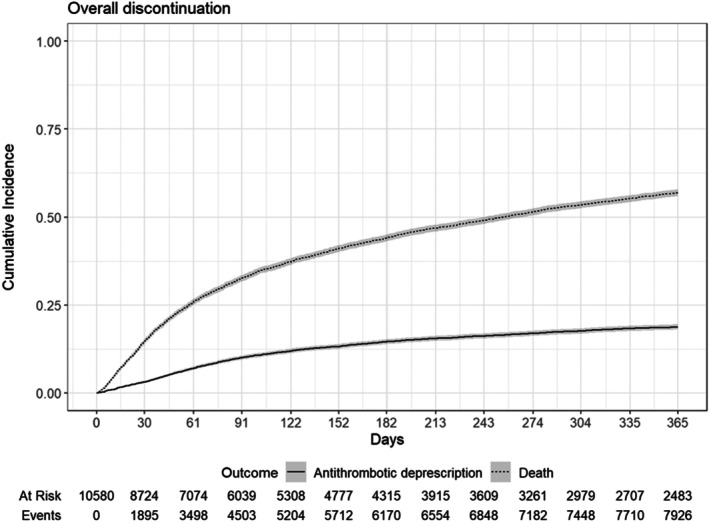
Cumulative incidence of discontinuation among individuals who were either ATT exposed at index. The 1‐year cumulative incidence was 19% (95% CI: 18%–20%). ATT, anti‐thrombotic therapy.

The 1‐year cumulative incidence of ATT discontinuation was 19% (95% CI: 18%–20%) with death as a competing risk (Figure 2, Table 3). The 1‐year incidence of discontinuation was highest among patients with gastrointestinal cancer (23% (95% CI: 14%–34%) for stage I, 25% (95% CI: 16–34) for stage II and 23% (95% CI: 20–26) for stage III) and stage IV urogenital cancers (24% (95% CI: 20–27)), while those with stage IV melanoma and other skin cancers (15% (95% CI: 7.5%–24%)), stage III respiratory and other intrathoracic organs (15% (95% CI: 14%–17%)) and stage III urogenital cancer (15% (95% CI: 9.7%–22%)) were significantly lower.

**TABLE 3:** One‐year cumulative incidence of ATT discontinuation in poor prognosis patients.

Variable	1‐year cumulative incidence of discontinuation (95% CI)
Overall	19% (18%–20%)
Sex	
Female	13% (12%–15%)
Male	14% (13%–15%)
Inclusion year	
2013	20% (18%–22%)
2014	19% (17%–21%)
2015	18% (16%–20%)
2016	20% (18%–22%)
2017	19% (17%–21%)
2018	19% (17%–21%)
2019	17% (15%–19%)
2020	19% (15%–22%)
2021	19% (12%–25%)
Cancer type	
Gastrointestinal, stage I	23% (14%–34%)***
Gastrointestinal, stage II	25% (16%–34%)***
Gastrointestinal, stage III	23% (20%–26%)***
Gastrointestinal, stage IV	21% (20%–22%)***
Gynaecological, stage IV	16% (11%–20%)***
Carcinoma of unknown primary, stage IV	22% (18%–27%)***
Melanoma and other skin cancers, stage IV	15% (7.5%–24%)***
Mesothelial and soft tissue, stage I	20% (9.0%–33%)***
Mesothelial and soft tissue, stage II	21% (7.1%–39%)***
Mesothelial and soft tissue, stage III	20% (10%–32%)***
Mesothelial and soft tissue, stage IV	20% (10%–32%)***
Respiratory and intrathoracic organs, stage III	15% (14%–17%)***
Respiratory and intrathoracic organs, stage IV	16% (14%–17%)***
Thyroid and other endocrine glands, stage IV	17% (6.0%–33%)***
Urogenital, stage III	15% (9.7%–22%)***
Urogenital, stage IV	24% (20%–27%)***
ATT indicator in the 1 year prior to index	
Any indicator	17% (15%–19%)
No indicator	19% (18%–20%)
Atrial fibrillation	18% (15%–21%)
No atrial fibrillation	15% (14%–16%)
ATE	14% (12%–17%)**
No ATE	19% (18%–20%)**
VTE	18% (15%–21%)
No VTE	19% (13%–14%)
ATT indicator in the 5 years prior to index	
Any indicator	18% (16%–19%)*
No indicator	20% (19%–21%)*
Atrial fibrillation	18% (16%–20%)***
No atrial fibrillation	15% (14%–16%)***
ATE	16% (15%–18%)**
No ATE	19% (18%–20%)**
VTE	18% (16%–20%)
No VTE	19% (18%–20%)
ATT indicator in the 10 years prior to index	
Any indicator	20% (19%–21%)*
No indicator	18% (16%–19%)*
Atrial fibrillation	18% (16%–20%)***
No atrial fibrillation	15% (14–16%)***
ATE	16% (15–18%)**
No ATE	19% (18–20%)**
VTE	17% (16–19%)
No VTE	19% (18%–20%)

Abbreviations: ATE, arterial thromboembolism; ATT, anti‐thrombotic therapy; VTE, venous thromboembolism.

*** *p* < 0.001.

** *p* < 0.01.

* *p* < 0.05.

Patients with ATT indicators in the 5 years before diagnosis were less likely to discontinue than those without (ATT indicator = 18%, 95% CI: 16%–19%, no ATT indicator = 20%, 95% CI: 19%–21%). An ATE in the 5 years prior to index was associated with a lower incidence of discontinuation than those without (16% (95% CI: 15%–18%) vs. 19% (95% CI: 18%–20%), *p* = 0.008), while atrial fibrillation in the previous 5 years was associated with a higher incidence of discontinuation compared to those without (18% (95% CI: 16%–20%) vs. 15% (95% CI: 14%–16%), *p* < 0.001). Year of inclusion and sex were not associated with discontinuation.

### Clinical outcomes

Within 1 year of follow‐up, 820 patients (3.2%) experienced a bleed and 1350 patients (5.2%) experienced a thromboembolism (64.5% VTE, 29.0% ATE, 12.3% MI) (Table 2). Seventy patients (0.2%) experienced both a bleed and a thromboembolism within 1 year of index.

**FIGURE 3:**
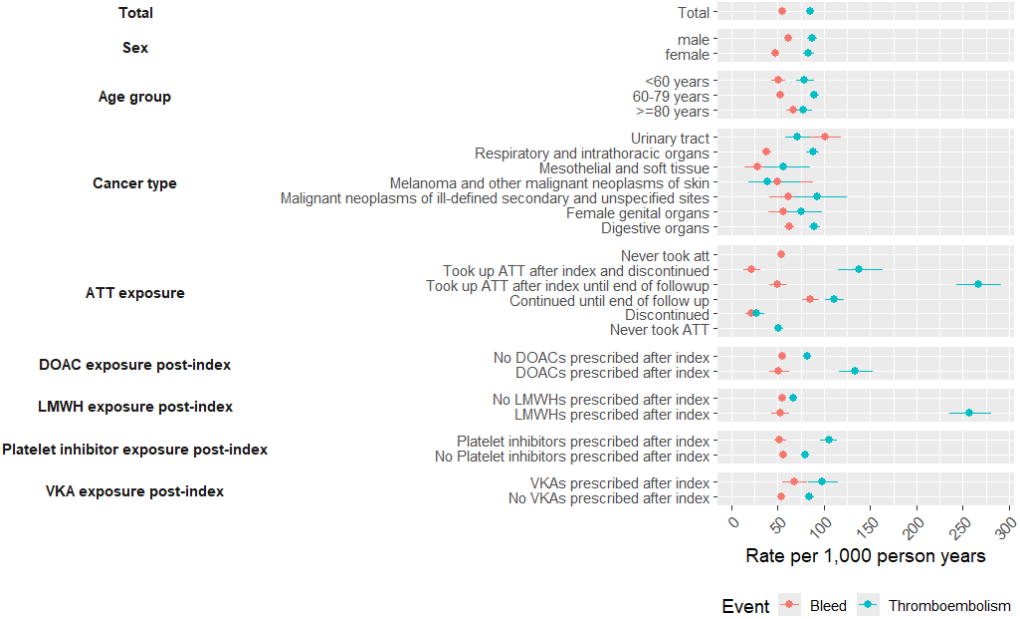
Rates of bleeding and thromboembolic events per 1000 person‐years (with 95% CI).

**FIGURE 4:**
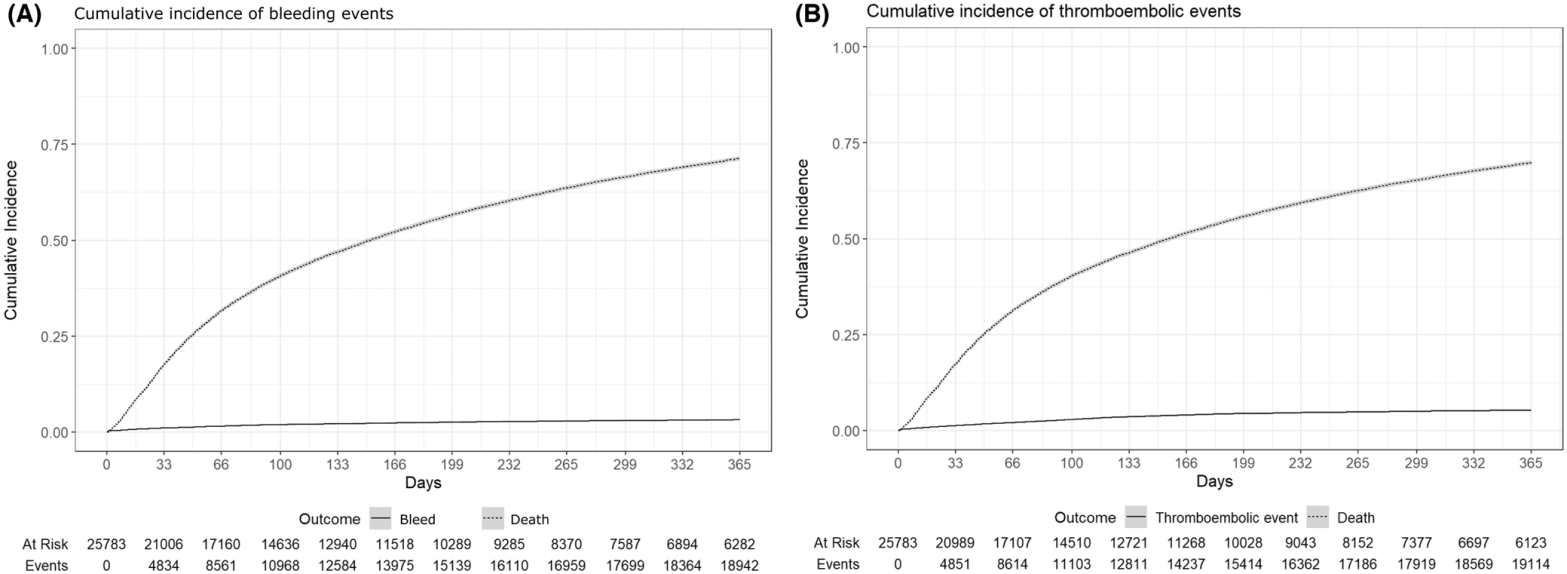
Cumulative incidence of bleeding (A) and thromboembolic events (B) within the cohort. The 1‐year cumulative incidence was 3.25 (95% CI: 3.0%–3.4%) for bleeding events and 5.3% (95% CI: 5.0%–5.6%) for thromboembolic events.

Rates of bleeding and thromboembolic events were 55.1/1000 PY (ranging between 21.1 and 101.0 according to subgroup) and 85.6/1000 PY (39.3–266.7). Rates of thromboembolisms were higher than for bleeding, except in patients with melanoma and urogenital cancer and for ATT unexposed at index (Figure 3). ATT use correlated with higher thromboembolism rates, especially in those initiated after diagnosis. LMWH users had the highest thromboembolism rate, followed by DOACs. Bleeding was lower in those who discontinued ATT. The overall 1‐year cumulative incidence of bleeding, accounting for the competing risk of death, was 3.2% (95% CI: 3.0%–3.4%) (Figure 4). Bleeding incidence was significantly associated with sex, with males experiencing a higher incidence than females (3.6% (95% CI: 3.3%–3.9%) vs. 2.6% (95% CI: 2.4%–3.0%)) (Table 4). However, neither ATT exposure at index nor inclusion year was significantly associated with the cumulative incidence of bleeding (*p* = 0.3 and 0.7 respectively).

**TABLE 4:** One‐year cumulative incidence of clinical outcomes.

Variable	Bleeds	Thromboembolism			
		All	ATE	MI	VTE
Overall	3.2% (3.0–3.4%)	5.3% (5.0–5.6%)	1.6% (1.4–1.7%)	0.45% (0.38–0.54%)	3.4% (3.2–3.7%)
Sex					
Female	2.6% (2.4–3.0%)***	5.1% (4.7–5.6%)			
Male	3.6% (3.3–3.9%)***	5.4% (5.1–5.8%)			
ATT usage at index					
ATT exposed at index	3.4% (3.0–3.8%)	4.7% (4.3–5.2%)**	1.8% (1.5–2.1%)	0.66% (0.50–0.86%)***	2.4% (2.1–2.8%)***
ATT unexposed at index	3.1% (2.8–3.4%)	5.6% (5.2–5.9%)**	1.5% (1.3–1.7%)	0.35% (0.27–0.45%)***	3.9% (3.6–4.2%)***

Abbreviations: ATE, arterial thromboembolism; ATT, anti‐thrombotic therapy; VTE, venous thromboembolism.

*** *p* < 0.001.

** *p* < 0.01.

In contrast, the 1‐year cumulative incidence of thromboembolism was higher at 5.3% (95% CI: 5.0%–5.6%) and varied by type of event (Figure 4). VTE was the most common form of thromboembolism (3.4%, 95% CI: 3.2%–3.7%) followed by ATE (1.6%, 95% CI: 1.4%–1.7%) and MI (0.45%, 95% CI: 0.38–0.54). ATT use at index was significantly associated with thromboembolism incidence, with a 1‐year cumulative incidence of 4.7% (95% CI: 4.3%–5.2%) among ATT exposed versus 5.6% (95% CI: 5.2%–5.9%) among unexposed. When stratifying by type of events, this relationship was most pronounced for VTE, with patients unexposed at index having a higher cumulative incidence compared to those who were exposed (3.9% (95% CI: 3.6%–4.2%) vs. 2.4% (95% CI: 2.1%–2.8%)). However, this relationship was reversed for MI, with those who were exposed at index experiencing a higher incidence of thromboembolic events (MI = 0.66% (95% CI: 0.50%–0.86%)) compared to those who were unexposed (MI = 0.35% (95% CI: 0.27%–0.45%)). Sex and year of inclusion were not associated with thromboembolism (*p* = 0.7, *p* = 0.2 respectively).

## DISCUSSION

In our analysis of 25 783 patients with poor prognosis cancer, totalling 8117 person‐years of follow‐up, we observed a median survival of 145 days. This suggests that the data likely reflect prescribing practices during the last year of life. At the time of cancer diagnosis, 32% of patients were exposed to ATT, and of those, 23% discontinued treatment before death. The 1‐year cumulative incidence of ATT discontinuation among all patients with poor prognosis cancer was 19%. We found a higher cumulative incidence of discontinuation in patients with urogenital and gastrointestinal cancers. Lower discontinuation rates were observed in those without a recent history of atrial fibrillation.

With 32% of patients receiving ATT at index, these data are similar to Danish registry findings (37.5%) and further challenge assertions that management of thrombosis and anti‐coagulation are beyond the remit of palliative care as a specialty.^18^, ^27^ Discontinuation of ATT prior to death was uncommon (19%), in keeping with other studies which report that most cancer patients continue ATT until end of life.^12^, ^18^, ^28^, ^29^ The decision whether to deprescribe or indeed initiate ATT near the end of life should involve balancing risks of bleeding versus thrombosis within the context of the patient's experience, values and preferences. Considering how to apply these data to clinical practice requires an appreciation of the nuances of bleeding and thromboembolism definitions, along with how they are recorded. Historically, bleeding definitions have been developed and refined by professional organisations such as the ISTH SSC, primarily to standardise outcome measures for clinical trials evaluating new anti‐coagulants and anti‐platelet agents.^30^ These focus on definitions of major and clinically relevant non‐major bleeds based on objective measures or clinical events and have also informed how they are coded on healthcare databases.^22^, ^23^, ^31^ Thromboembolic events are usually recorded according to type of event (DVT, PE, ATE, MI, etc.), with limited detail of severity beyond capturing fatal events.

Such outcomes are of limited utility in patients with terminal illness for two main reasons. First, in patients nearing the end of life, there is a greater emphasis on quality of life and outcome measures pertaining to survival are of less importance. Second, while these results are unlikely to record minor bleeds such as bruising and epistaxis, their impact on patients' quality of life can be substantial and plays a significant role in clinical decision‐making.^32^, ^33^ Similarly, a binary recording of a thromboembolic event does not capture clot severity, let alone the clinical or psychological impact.

These results should be viewed in the context of the recognised strengths and limitations of large cohort studies from patient databases. One strength lies in using a nationwide e‐cohort to represent the population of Wales, drawing from multiple high‐quality data sources to follow patients experiencing end‐of‐life care. Previous studies have focussed solely on patients with stage IV cancers, using the presence of metastases as a proxy for poor prognosis.^34^, ^35^ We focus on all patients diagnosed with a poor prognosis cancer regardless of disease stage, thus providing a more accurate reflection of the population of interest. This is more likely to reflect real‐world practice since clinicians will already have identified those most likely to need a proactive approach to advance care planning. This cohort had a median survival of 145 days (95% CI: 142–148) suggesting this selection criteria are valid.

There are also limitations to this study. First, our prescription data are limited to those prescriptions supplied by a GP; we are unable to account for those provided by alternative sources, such as secondary or hospice care. Where patients spend significant periods of time as hospital or hospice in‐patients, the absence of ATT prescribing in primary care may infer an overestimation of the discontinuation rate. Second, there are several challenges associated with defining ATT discontinuation and the date thereof. ATT discontinuation was defined as the final prescription received by a patient, plus an additional 2 months, which captures 95% of repeat prescriptions in the Welsh population. Additionally, variation in prescription length is not accounted for by this assumption, which may be of relevance to end of life care where medication is reviewed more frequently. Patients may also discontinue ATT before the end of their prescription or even not take their medication at all. This method also does not account for dose reductions and patients who discontinue and then restart treatment, missing out on dynamic changes in ATT exposure. We were unable to ascertain the severity of clinical outcome from ICD‐10 codes alone, limiting our study to a binary classification of whether our patient experienced an event or not. This may oversimplify our results, by not accounting for the range of severity in real‐life outcomes. Moreover, these data do not capture events recorded in primary care and may therefore underrepresent these outcomes, as patients in the very last phase of life may not be referred to the hospital anymore. Finally, our study focuses on cancer patients with a poor expected outcome; however, this may not be representative of all cancer patients in their final 12 months of life.^36^

This nationwide cohort study corroborates previous reports that the majority of terminally ill cancer patients receiving ATT continue these medicines until death. This reinforces the need for a decision support tool to facilitate shared decision‐making and help rationalise ATT in the advanced cancer setting, which is currently being developed within the Serenity project.^19^

## AUTHOR CONTRIBUTIONS

EKK, GJG, JG, MS, SCC and SJA were involved in the design of the study. SJA performed the data extraction and analysis and is responsible for the integrity of the data and accuracy of the data analysis. SJA and SN drafted the initial version of the manuscript. All authors contributed to the interpretation of the data, critically revised the manuscript and approved the final version of the manuscript. SN and EK are Co‐Cis of the SERENITY programme.

## CONFLICT OF INTEREST STATEMENT

FAK has received research support from Bayer, BMS, BSCI, AstraZeneca, MSD, Leo Pharma, Actelion, Farm‐X, angiodynamics, The Netherlands Organisation for Health Research and Development, The Dutch Thrombosis Foundation, The Dutch Heart Foundation and the Horizon Europe Program, all outside this work and paid to his institution. No other authors have conflicts of interest to declare.

## ETHICS APPROVAL STATEMENT

Approval for the use of anonymised data in this study, provisioned within the Secure Anonymised Information Linkage (SAIL) Databank, was granted by an independent Information Governance Review Panel (IGRP) under project 1376. The IGRP has a membership comprised of senior representatives from the British Medical Association (BMA), the National Research Ethics Service (NRES), Public Health Wales and Digital Health and Care Wales (DHCW). The usage of additional data was granted by each respective data owner. The SAIL Databank is compliant with General Data Protection Regulations (GDPR) and the UK Data Protection Act.

## Supporting information


Data S1.

## Data Availability

The data used in this study are available in the SAIL Databank at Swansea University, Swansea, United Kingdom, but as restrictions apply, they are not publicly available. All proposals to use SAIL data are subject to review by an independent Information Governance Review Panel (IGRP). Before any data can be accessed, approval must be given by the IGRP. The IGRP carefully considers each project to ensure the proper and appropriate use of SAIL data. When access has been granted, it is gained through a privacy‐protecting trusted research environment (TRE) and remote access system referred to as the SAIL Gateway. SAIL has established an application process to be followed by anyone who would like to access data via SAIL at https://saildatabank.com/data/apply‐to‐work‐with‐the‐data/. The scripts used for this manuscript are publicly available in the SAIL GIT repository found at https://github.com/SwanseaUniversityDataScience/SERENITY.
