# Supplementary material for: Anti‐thrombotic therapy in patients with cancer at the end of life: A cohort study using population‐linked routinely collected data
Source: Br J Haematol. 2025 Sep 1;207(5):2080–90. doi: 10.1111/bjh.70032 (PMC12624168; doi:10.1111/bjh.70032)
Supplement: Supplementary file 1 — Data S1. [file BJH-207-2080-s001.docx]

Supplementary material

Contents

[S1.0 Clinical codes 1](#_Toc199251459)

[S1.1 Cancer Clinical codes 1](#_Toc199251460)

[S1.2 ATT Prescription and discontinuation clinical codes 3](#_Toc199251461)

[S1.3 Bleeding clinical codes 8](#_Toc199251462)

[S1.4 Thromboembolism clinical codes 9](#_Toc199251463)

[S2.0 Cohort 10](#_Toc199251464)

[S2.1 List of SAIL data sources used in this publication 10](#_Toc199251465)

[S2.2 Cohort cleaning steps 10](#_Toc199251466)

[S2.3 Prescription interval 10](#_Toc199251467)

[S3.0 Survival analysis 11](#_Toc199251468)

[S3.1 Survival probability 11](#_Toc199251469)

[S3.2 Cumulative incidence 14](#_Toc199251470)

# S1.0 Clinical codes

## S1.1 Cancer Clinical codes

The following plot and table lists all the cancer types that were included, and their Net 1 year survival % (and 95% CI) in the UK. All data were taken from Welsh Government statistics found at https://www.gov.wales/cancer-survival-wales-2002-2020.


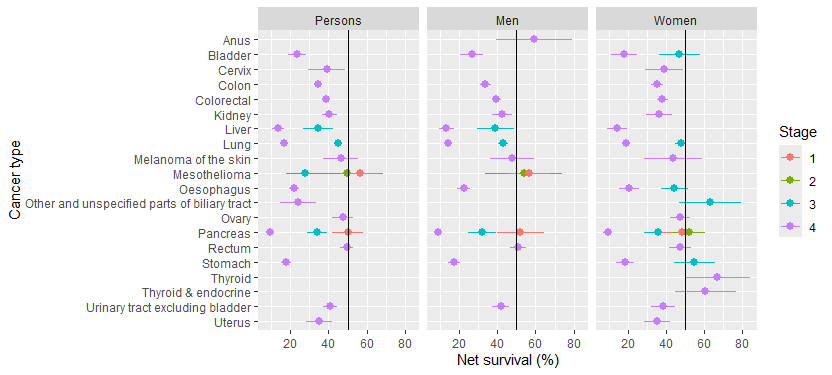


|  |  |  | **Net 1 year survival % (95% CI) for cancer stages 1-4** | | | |
| --- | --- | --- | --- | --- | --- | --- |
| **Cancer type** | **ICD-10 code** | **Sex** | **Stage 1** | **Stage 2** | **Stage 3** | **Stage 4** |
| Anus | C21 | Men | NA | NA | NA | 59.34 (39.5-79.19) |
| Bladder | C67 | Persons | NA | NA | NA | 23.66 (19.09-28.23) |
|  |  | Men | NA | NA | NA | 26.67 (20.73-32.61) |
|  |  | Women | NA | NA | 47.03 (36.37-57.69) | 18.18 (11.33-25.02) |
| Cancer of unknown primary | C76-C80 | Persons | NA | NA | NA | 6.27 (9.72-13.16) |
| Cervix | C53 | Persons | NA | NA | NA | 39.04 (29.23-48.86) |
|  |  | Women | NA | NA | NA | 39.04 (29.23-48.86) |
| Colon | C18 | Persons | NA | NA | NA | 34.42 (32.3-36.54) |
|  |  | Men | NA | NA | NA | 33.84 (30.96-36.72) |
|  |  | Women | NA | NA | NA | 35.1 (31.98-38.22) |
| Colorectal | C18-C20 | Persons | NA | NA | NA | 38.92 (37.1-40.75) |
|  |  | Men | NA | NA | NA | 39.52 (37.09-41.95) |
|  |  | Women | NA | NA | NA | 38.12 (35.35-40.89) |
| Gallbladder | C23 | Persons | NA | NA | NA | 26.15 (17.58-34.73) |
|  |  | Women | NA | NA | NA | 25.54 (15.81-35.27) |
| Kidney | C64 | Persons | NA | NA | NA | 40.55 (36.46-44.63) |
|  |  | Men | NA | NA | NA | 42.55 (37.53-47.57) |
|  |  | Women | NA | NA | NA | 36.41 (29.48-43.34) |
| Liver | C22 | Persons | NA | NA | 34.65 (26.72-42.58) | 13.74 (10.58-16.9) |
|  |  | Men | NA | NA | 39.1 (29.64-48.56) | 13.41 (9.49-17.34) |
|  |  | Women | NA | NA | NA | 14.26 (9.02-19.51) |
| Lung | C33-C34 | Persons | NA | NA | 45.24 (43.26-47.23) | 16.67 (15.68-17.67) |
|  |  | Men | NA | NA | 43.02 (40.29-45.75) | 14.28 (12.99-15.56) |
|  |  | Women | NA | NA | 47.7 (44.82-50.59) | 19.39 (17.86-20.92) |
| Melanoma of the skin | C43 | Persons | NA | NA | NA | 46.43 (37.19-55.67) |
|  |  | Men | NA | NA | NA | 47.88 (36.44-59.31) |
|  |  | Women | NA | NA | NA | 43.67 (28.33-59) |
| Mesothelioma | C45 | Persons | 56.33 (46.19-66.48) | 49.89 (31.44-68.34) | 27.75 (17.68-37.82) | NA |
|  |  | Men | 56.66 (45.65-67.66) | 53.78 (33.82-73.73) | NA | NA |
| Oesophagus | C15 | Persons | NA | NA | NA | 22.07 (19.29-24.85) |
|  |  | Men | NA | NA | NA | 22.51 (19.23-25.79) |
|  |  | Women | NA | NA | 44.39 (37.36-51.42) | 20.88 (15.69-26.06) |
| Other and unspecified parts of biliary tract | C24 | Persons | NA | NA | NA | 24.17 (14.74-33.59) |
|  |  | Women | NA | NA | 62.93 (46.68-79.17) | NA |
| Ovary | C56 | Persons | NA | NA | NA | 47.39 (42.02-52.77) |
|  |  | Women | NA | NA | NA | 47.39 (42.02-52.77) |
| Pancreas | C25 | Persons | 50.04 (41.79-58.28) | NA | 34 (28.77-39.24) | 9.33 (7.78-10.88) |
|  |  | Men | 52.09 (39.95-64.24) | NA | 32.21 (24.85-39.56) | 9.16 (7.05-11.27) |
|  |  | Women | 48.23 (37.14-59.33) | 51.83 (43.25-60.4) | 35.76 (28.38-43.14) | 9.52 (7.25-11.79) |
| Rectum | C19-C20 | Persons | NA | NA | NA | 49.63 (46.22-53.03) |
|  |  | Men | NA | NA | NA | 50.75 (46.54-54.97) |
|  |  | Women | NA | NA | NA | 47.47 (41.72-53.21) |
| Stomach | C16 | Persons | NA | NA | NA | 17.89 (15.26-20.51) |
|  |  | Men | NA | NA | NA | 17.47 (14.31-20.63) |
|  |  | Women | NA | NA | 54.84 (44.07-65.6) | 18.76 (14.07-23.45) |
| Thyroid | C73 | Women | NA | NA | NA | 66.81 (49.88-83.73) |
| Thyroid & endocrine | C73-C75 | Women | NA | NA | NA | 60.64 (44.52-76.75) |
| Urinary tract excluding bladder | C64-C66, C68 | Persons | NA | NA | NA | 40.66 (36.9-44.41) |
|  |  | Men | NA | NA | NA | 41.74 (37.1-46.39) |
|  |  | Women | NA | NA | NA | 38.51 (32.15-44.87) |
| Uterus | C54 | Persons | NA | NA | NA | 35.17 (28.41-41.93) |
|  |  | Women | NA | NA | NA | 35.17 (28.41-41.93) |

## S1.2 ATT Prescription and discontinuation clinical codes

Read codes for ATT prescriptions

| **code** | **type** | **category** | **description** |
| --- | --- | --- | --- |
| 44vh. | ATT prescription | DOAC | Plasma rivaroxaban LEVEL |
| 8IHG. |  |  | Apixaban declined |
| 8IHH. |  |  | Rivaroxaban declined |
| bs4.. |  |  | DABIGATRAN ETEXILATE |
| bs41. |  |  | PRADAXA 75mg capsules |
| bs42. |  |  | PRADAXA 110mg capsules |
| bs43. |  |  | PRADAXA 150mg capsules |
| bs4x. |  |  | DABIGATRAN ETEXILATE 150mg capsules |
| bs4y. |  |  | DABIGATRAN ETEXILATE 110mg capsules |
| bs4z. |  |  | DABIGATRAN ETEXILATE 75mg capsules |
| bs6.. |  |  | RIVAROXABAN |
| bs61. |  |  | XARELTO 10mg tablets |
| bs62. |  |  | XARELTO 15mg tablets |
| bs63. |  |  | XARELTO 20mg tablets |
| bs64. |  |  | XARELTO 2.5mg tablets |
| bs6w. |  |  | RIVAROXABAN 2.5mg tablets |
| bs6x. |  |  | RIVAROXABAN 20mg tablets |
| bs6y. |  |  | RIVAROXABAN 15mg tablets |
| bs6z. |  |  | RIVAROXABAN 10mg tablets |
| bs7.. |  |  | APIXAN |
| bs71. |  |  | ELIQUIS 2.5mg tablets |
| bs72. |  |  | APIXABAN 2.5mg tablets |
| bs73. |  |  | ELIQUIS 5mg tablets |
| bs74. |  |  | APIXABAN 5mg tablets |
| bs8.. |  |  | EDOXABAN |
| bs81. |  |  | LIXIANA 15mg tablets |
| bs82. |  |  | LIXIANA 30mg tablets |
| bs83. |  |  | LIXIANA 60mg tablets |
| bs84. |  |  | EDOXABAN 15mg tablets |
| bs85. |  |  | EDOXABAN 30mg tablets |
| bs86. |  |  | EDOXABAN 60mg tablets |
| 1Z340 | deprescription indicator |  | Apixaban adverse reaction |
| 1Z341 |  |  | Rivaroxaban adverse reaction |
| 1Z430 |  |  | Rivaroxaban allergy |
| 1Z431 |  |  | Apixaban allergy |
| 8I7R. |  |  | Dabigatran not tolerated |
| 8I7Z. |  |  | Apixaban not tolerated |
| 8I7a. |  |  | Rivaroxaban not tolerated |
| br2A. | ATT prescription | Low molecular weight heparins | DALTEPARIN SODIUM 100000iu/4mL injection vial |
| br2C. |  |  | DALTEPARIN SODIUM 10,000iu/0.4mL prefilled syringe |
| br2D. |  |  | DALTEPARIN SODIUM 12500iu/0.5mL prefilled syringe |
| br2E. |  |  | DALTEPARIN SODIUM 18000iu/0.72mL prefilled syringe |
| br2I. |  |  | DALTEPARIN SODIUM 15000iu/0.6mL prefilled syringe |
| br2L. |  |  | DALTEPARIN SODIUM 7500iu/0.3mL prefilled syringe |
| br2l. |  |  | DALTEPARIN SODIUM 10000iu/1mL subcutaneous injection |
| br2m. |  |  | DALTEPARIN SODIUM 10000iu/4mL subcutaneous injection |
| br2n. |  |  | DALTEPARIN SODIUM 12500iu/mL prefilled syringe 0.2mL |
| br2o. |  |  | DALTEPARIN SODIUM 25000iu/mL prefilled syringe 0.2mL |
| br2z. |  |  | DALTEPARIN SODIUM 10000iu/1mL prefilled syringe |
| br6.. |  |  | ENOXAPARIN |
| br61. |  |  | ENOXAPARIN 20mg/0.2mL prefilled syringe |
| br62. |  |  | ENOXAPARIN 40mg/0.4mL prefilled syringe |
| br63. |  |  | CLEXANE 20mg/0.2mL prefilled syringe |
| br64. |  |  | CLEXANE 40mg/0.4mL prefilled syringe |
| br65. |  |  | ENOXAPARIN 60mg/0.6mL prefilled syringe |
| br66. |  |  | ENOXAPARIN 80mg/0.8mL prefilled syringe |
| br67. |  |  | ENOXAPARIN 100mg/1mL prefilled syringe |
| br68. |  |  | CLEXANE 60mg/0.6mL prefilled syringe |
| br69. |  |  | CLEXANE 80mg/0.8mL prefilled syringe |
| br6A. |  |  | CLEXANE 100mg/1mL prefilled syringe |
| br6B. |  |  | CLEXANE 120mg/0.8mL prefilled syringe |
| br6C. |  |  | CLEXANE 150mg/1mL prefilled syringe |
| br6D. |  |  | CLEXANE 300mg/3mL solution for injection |
| br6x. |  |  | ENOXAPARIN 300mg/3mL solution for injection |
| br6y. |  |  | ENOXAPARIN 120mg/0.8mL prefilled syringe |
| br6z. |  |  | ENOXAPARIN 150mg/1mL prefilled syringe |
| br7.. |  |  | TINZAPARIN SODIUM |
| br71. |  |  | INNOHEP 3500iu(anti Xa)/0.3mL subcutaneous injection in syringe |
| br72. |  |  | INNOHEP 5000iu(anti Xa)/0.5mL subcutaneous injection ampoules |
| br73. |  |  | LOGIPARIN 2500iu/0.21mL subcutaneous injection in syringe |
| br74. |  |  | LOGIPARIN 3500iu/0.3mL subcutaneous injection in syringe |
| br75. |  |  | LOGIPARIN 4500iu/0.39mL subcutaneous injection in syringe |
| br76. |  |  | TINZAPARIN SODIUM 2500iu/0.21mL subcutaneous injection in syringe |
| br77. |  |  | TINZAPARIN SODIUM 3500iu(anti Xa)/0.3mL subcutaneous injection insyringe |
| br78. |  |  | TINZAPARIN SODIUM 4500iu/0.39mL subcutaneous injection in syringe |
| br79. |  |  | TINZAPARIN SODIUM 5000iu(anti Xa)/0.5mL subcutaneous injection ampoules |
| br7A. |  |  | INNOHEP 40,000iu(anti Xa)/2mL subcutaneous injection vials |
| br7B. |  |  | TINZAPARIN SODIUM 40,000iu(anti Xa)/2mL subcutaneous injection vials |
| br7B. |  |  | TINZAPARIN SODIUM 40000iu(anti Xa)/2mL subcutaneous injection vials |
| br7C. |  |  | TINZAPARIN SODIUM 5000iu(anti Xa)/5mL injection ampoules |
| br7D. |  |  | INNOHEP 5000iu(anti Xa)/5mL injection ampoules |
| br7E. |  |  | TINZAPARIN SODIUM 10,000iu(anti Xa)/0.5mL prefilled syringe |
| br7E. |  |  | TINZAPARIN SODIUM 10000iu(anti Xa)/0.5mL prefilled syringe |
| br7F. |  |  | TINZAPARIN SODIUM 14,000iu(anti Xa)/0.7mL prefilled syringe |
| br7F. |  |  | TINZAPARIN SODIUM 14000iu(anti Xa)/0.7mL prefilled syringe |
| br7G. |  |  | TINZAPARIN SODIUM 18,000iu(anti Xa)/0.9mL prefilled syringe |
| br7G. |  |  | TINZAPARIN SODIUM 18000iu(anti Xa)/0.9mL prefilled syringe |
| br7H. |  |  | INNOHEP 10,000iu(anti Xa)/0.5mL prefilled syringe |
| br7J. |  |  | INNOHEP 14,000iu(anti Xa)/0.7mL prefilled syringe |
| br7K. |  |  | INNOHEP 18,000iu(anti Xa)/0.9mL prefilled syringe |
| br7L. |  |  | TINZAPARIN SODIUM 3500iu(anti Xa)/0.35mL prefilled syringe |
| br7M. |  |  | TINZAPARIN SODIUM 4500iu(anti Xa)/0.45mL prefilled syringe |
| br7N. |  |  | INNOHEP 3500iu(anti Xa)/0.35mL prefilled syringe |
| br7O. |  |  | INNOHEP 4500iu(anti Xa)/0.45mL prefilled syringe |
| br7P. |  |  | TINZAPARIN SODIUM 20,000iu(anti Xa)/2mL injection vial |
| br7P. |  |  | TINZAPARIN SODIUM 20000iu(anti Xa)/2mL injection vial |
| br7Q. |  |  | INNOHEP 20,000iu(anti Xa)/2mL injection vial |
| br7R. |  |  | INNOHEP 2500iu(anti Xa)/0.25mL prefilled syringe |
| br7S. |  |  | TINZAPARIN SODIUM 2500iu(anti Xa)/0.25mL prefilled syringe |
| br7T. |  |  | INNOHEP 8000iu(anti Xa)/0.4mL prefilled syringe |
| br7U. |  |  | INNOHEP 12,000iu(anti Xa)/0.6mL prefilled syringe |
| br7V. |  |  | INNOHEP 16,000iu(anti Xa)/0.8mL prefilled syringe |
| br7W. |  |  | TINZAPARIN SODIUM 8000iu(anti Xa)/0.4mL prefilled syringe |
| br7X. |  |  | TINZAPARIN SODIUM 12,000iu(anti Xa)/0.6mL prefilled syringe |
| br7X. |  |  | TINZAPARIN SODIUM 12000iu(anti Xa)/0.6mL prefilled syringe |
| br7Y. |  |  | TINZAPARIN SODIUM 16,000iu(anti Xa)/0.8mL prefilled syringe |
| br7Y. |  |  | TINZAPARIN SODIUM 16000iu(anti Xa)/0.8mL prefilled syringe |
| brD.. |  |  | FONDAPARINUX |
| brD1. |  |  | FONDAPARINUX SODIUM 2.5mg/0.5mL prefilled syringe |
| brD2. |  |  | FONDAPARINUX SODIUM 5mg/0.4mL prefilled syringe |
| brD3. |  |  | FONDAPARINUX SODIUM 7.5mg/0.6mL prefilled syringe |
| brD4. |  |  | FONDAPARINUX SODIUM 10mg/0.8mL prefilled syringe |
| brD5. |  |  | ARIXTRA 5mg/0.4mL prefilled syringe |
| brD6. |  |  | ARIXTRA 7.5mg/0.6mL prefilled syringe |
| brD7. |  |  | ARIXTRA 10mg/0.8mL prefilled syringe |
| brD8. |  |  | FONDAPARINUX SODIUM 1.5mg/0.3mL prefilled syringe |
| brD9. |  |  | ARIXTRA 2.5mg/0.5mL prefilled syringe |
| brDZ. |  |  | ARIXTRA 1.5mg/0.3mL prefilled syringe |
| 67I8. | ATT prescription | Platelet inhibitors | Advice about taking aspirin |
| 8B3T. |  |  | Over the counter aspirin therapy |
| 8B63. |  |  | Aspirin prophylaxis |
| 8B63. |  |  | Aspirin prophylaxis - IHD |
| 8B6P. |  |  | Clopidogrel prophylaxis |
| 8BG5. |  |  | Aspirin indicated |
| 8BG9. |  |  | Clopidogrel indicated |
| bu2.. |  |  | ASPIRIN [ANTIPLATELET] |
| bu21. |  |  | ASPIRIN 100mg effervescent tablets |
| bu22. |  |  | PLATET 100mg effervescent tablets |
| bu23. |  |  | ASPIRIN 75mg dispersible tablets |
| bu24. |  |  | *ANGETTES 75mg tablets |
| bu25. |  |  | *ASPIRIN 75mg tablets |
| bu26. |  |  | PLATET 300mg effervescent tablets |
| bu27. |  |  | ASPIRIN 300mg effervescent tablets |
| bu28. |  |  | *DISPRIN CV 100mg m/r tablets |
| bu29. |  |  | *ASPIRIN 100mg m/r tablets |
| bu2A. |  |  | NU-SEALS ASPIRIN 75mg e/c tablets |
| bu2B. |  |  | ASPIRIN 75mg e/c tablets |
| bu2C. |  |  | *POSTMI 300mg e/c tablets |
| bu2D. |  |  | POSTMI 75mg dispersible tablets |
| bu2E. |  |  | *POSTMI 75mg e/c tablets |
| bu2F. |  |  | *CAPRIN 75mg e/c tablets |
| bu2G. |  |  | NU-SEALS CARDIO 75 e/c tablets |
| bu2H. |  |  | *ENPRIN 75mg e/c tablets |
| bu2I. |  |  | ASPIRIN 162.5mg m/r capsules |
| bu2J. |  |  | CASPAC XL 162.5mg m/r capsules |
| bu2K. |  |  | MICROPIRIN 75mg e/c tablets |
| bu2a. |  |  | *DISPRIN CV 300mg m/r tablets |
| bu2b. |  |  | *ASPIRIN 300mg m/r tablets |
| bu2c. |  |  | ASPIRIN 75mg soluble tablets |
| bu2d. |  |  | FLAMASACARD 162.5mg m/r capsules |
| bu4.. |  |  | DIPYRIDAMOLE+ASPIRIN |
| bu41. |  |  | DIPYRIDAMOLE+ASPIRIN 200mg/25mg m/r capsules |
| bu42. |  |  | ASASANTIN RETARD m/r capsules |
| bu43. |  |  | MOLITA 200mg/25mg m/r capsules |
| bu5.. |  |  | CLOPIDOGREL |
| bu51. |  |  | CLOPIDOGREL 75mg tablets |
| bu52. |  |  | PLAVIX 75mg tablets |
| bu53. |  |  | PLAVIX 300mg tablets |
| bu54. |  |  | CLOPIDOGREL 300mg tablets |
| bu55. |  |  | GREPID 75mg tablets |
| buA.. |  |  | PRASUGREL |
| buA1. |  |  | EFIENT 5mg tablets |
| buA2. |  |  | PRASUGREL 5mg tablets |
| buA3. |  |  | EFIENT 10mg tablets |
| buA4. |  |  | PRASUGREL 10mg tablets |
| buB.. |  |  | TICAGRELOR |
| buB1. |  |  | BRILIQUE 90mg tablets |
| buBz. |  |  | TICAGRELOR 90mg tablets |
| di1.. |  |  | ASPIRIN [CENTRAL NERVOUS SYSTEM USE] |
| di11. |  |  | ASPIRIN [CNS] 300mg tablets |
| di12. |  |  | ASPIRIN [CNS] 300mg dispersible tablets |
| di13. |  |  | ASPIRIN 75mg dispersible tablets |
| di14. |  |  | *ASPERGUM 227mg chewing gum |
| di15. |  |  | *CLARADIN 300mg tablets DISCONTINUED |
| di16. |  |  | *LABOPRIN 300mg tablets |
| di17. |  |  | *PAYNOCIL 600mg tablets |
| di18. |  |  | SOLPRIN 300mg dispersible tablets DISCONTINUED |
| di19. |  |  | *ASPIRIN 500mg m/r tablets |
| di1a. |  |  | *CAPRIN 324mg e/c tablets |
| di1b. |  |  | *LEVIUS 500mg m/r tablets DISCONTINUED |
| di1c. |  |  | NU-SEALS ASPIRIN 300mg e/c tablets |
| di1d. |  |  | NU-SEALS ASPIRIN 600mg e/c tablets |
| di1e. |  |  | *PALAPRIN FORTE 600mg tablets |
| di1f. |  |  | ASPIRIN 300mg e/c tablets |
| di1g. |  |  | *ASPIRIN 600mg e/c tablets |
| di1h. |  |  | *ASPIRIN 324mg e/c tablets |
| di1i. |  |  | *ASPIRIN 600mg tablets |
| di1j. |  |  | *LABOPRIN DL 900mg sachets |
| di1k. |  |  | *CAPRIN 300mg e/c tablets DISCONTINUED |
| di1m. |  |  | ASPIRIN 300mg soluble tablets |
| di1n. |  |  | ASPIRIN 300mg suppositories |
| di1o. |  |  | ASPIRIN 150mg suppositories |
| di1r. |  |  | DISPRIN 300mg dispersible tablets |
| dia5. |  |  | ASPIRIN+CODEINE 400mg/8mg dispersible tablets |
| diaO. |  |  | ASPIRIN+CODEINE PHOSPHATE 500mg/8mg dispersible tablets |
| dl1b. |  |  | ASPIRIN+METOCLOPRAMIDE 900mg/10mg/sachet powder |
| j1... |  |  | ASPIRIN AND THE SALICYLATES |
| j11.. |  |  | ASPIRIN [MUSCULOSKELETAL USE] |
| j111. |  |  | ASPIRIN 300mg tablets |
| j112. |  |  | ASPIRIN 300mg dispersible tablets |
| j13.. |  |  | CHOLINE MAGNES. TRISALICYLATE |
| j131. |  |  | *TRILISATE 500mg tablets |
| j13z. |  |  | CHOLINE MAGNES. TRISAL 500mg tablets |
| j14.. |  |  | SALSALATE |
| j141. |  |  | *DISALCID 500mg capsules |
| j14z. |  |  | *SALSALATE 500mg capsules |
| j15.. |  |  | SODIUM SALICYLATE |
| j151. |  |  | SODIUM SALICYLATE 250mg/5mL mixture |
| j152. |  |  | SODIUM SALICYLATE strong mixture |
| 14LK. | deprescription indicator |  | H/O: aspirin allergy |
| 1Z32. |  |  | Prasugrel adverse reaction |
| 1Z33. |  |  | Ticagrelor adverse reaction |
| 1Z41. |  |  | Prasugrel allergy |
| 1Z42. |  |  | Ticagrelor allergy |
| 8B6P1 |  |  | Clopidogrel therapy stopped |
| 8I70. |  |  | Aspirin not tolerated |
| 8I7X. |  |  | Prasugrel not tolerated |
| 8I7Y. |  |  | Ticagrelor not tolerated |
| TJ53. |  |  | Adverse reaction to aspirin |
| U6051 |  |  | [X] Adverse reaction to aspirin |
| ZV148 |  |  | [V]Personal history of aspirin allergy |
| 66Q.. | ATT prescription | VKA | Warfarin monitoring |
| 66Q1. |  |  | Initial warfarin assessment |
| 66Q2. |  |  | Follow-up warfarin assessment |
| 66Q4. |  |  | Warfarin dose changed |
| 66Q6. |  |  | Warfarin therapy started |
| 66Q7. |  |  | Target international normalised ratio |
| 66Q70 |  |  | INR (international normalised ratio) target range |
| 66Q8. |  |  | International normalised ratio deviation from target |
| 66Q80 |  |  | International normalised ratio above target range |
| 66Q9. |  |  | Warfarin dose unchanged |
| 66QA. |  |  | Warfarin treatment plan |
| 66QB. |  |  | Annual warfarin assessment |
| 66QC. |  |  | Anticoagulation monitoring - secondary care |
| 66QD. |  |  | Anticoagulation monitoring - primary care |
| 66QF. |  |  | Slow induction of warfarin therapy |
| 66QG. |  |  | International normalised ratio derived warfarin dose |
| 66QH. |  |  | Warfarin daily dose |
| 66QZ. |  |  | Warfarin monitoring NOS |
| 8B610 |  |  | Warfarin anticoagulation prophylaxis |
| 8BG7. |  |  | Warfarin indicated |
| 8BMJ1 |  |  | Dispensing review of use of warfarin |
| 8BPF. |  |  | Requires lifelong warfarin therapy |
| 8HHW. |  |  | Referral for warfarin monitoring |
| bs1.. |  |  | WARFARIN SODIUM |
| bs11. |  |  | MAREVAN 1mg tablets |
| bs12. |  |  | MAREVAN 3mg tablets |
| bs13. |  |  | MAREVAN 5mg tablets |
| bs14. |  |  | *WARFARIN WBP 1mg tablets |
| bs15. |  |  | *WARFARIN WBP 3mg tablets |
| bs16. |  |  | *WARFARIN WBP 5mg tablets |
| bs17. |  |  | WARFARIN SODIUM 1mg tablets |
| bs18. |  |  | WARFARIN SODIUM 3mg tablets |
| bs19. |  |  | WARFARIN SODIUM 5mg tablets |
| bs1A. |  |  | WARFARIN SODIUM 0.5mg tablets |
| bs1B. |  |  | MAREVAN 0.5mg tablets |
| bs1C. |  |  | WARFARIN SODIUM 1mg/1mL oral suspension |
| bs2.. |  |  | ACENOCOUMAROL |
| bs21. |  |  | SINTHROME 1mg tablets |
| bs22. |  |  | *SINTHROME 4mg tablets |
| bs23. |  |  | ACENOCOUMAROL 1mg tablets |
| bs24. |  |  | *NICOUMALONE 4mg tablets DISCONTINUED |
| 14LP. | deprescription indicator |  | H/O: warfarin allergy |
| 66Q5. |  |  | Warfarin therapy stopped |
| 8I71. |  |  | Warfarin not tolerated |
| TJ421 |  |  | Adverse reaction to warfarin sodium |
| U6042 |  |  | [X] Adverse reaction to warfarin sodium |

## S1.3 Bleeding clinical codes

Below are the ICD-10 codes for major and non-major, clinically relevant bleeding used to identify clinical outcomes in the cohort.

| **Code** | **Description** |
| --- | --- |
| D62. | Acute posthaemorrhagic anaemia |
| D683 | Haemorrhagic disorder due to circulating anticoagulants |
| H113 | Conjunctival haemorrhage |
| H210 | Hyphaema |
| H313 | Choroidal haemorrhage and rupture |
| H356 | Retinal haemorrhage |
| H431 | Vitreous haemorrhage |
| H450 | Vitreous haemorrhage in diseases classified elsewhere |
| H922 | Otorrhagia |
| I230 | Haemopericardium as current complication following acute myocardial infarction |
| I312 | Haemopericardium, not elsewhere classified |
| I60. | Subarachnoid haemorrhage |
| I61. | Intracerebral haemorrhage |
| I62. | Other nontraumatic intracranial haemorrhage |
| I850 | Oesophageal varices with bleeding |
| I983 | Oesophageal varices with bleeding in diseases classified elsewhere |
| J942 | Haemothorax |
| K226 | Gastro-oesophageal laceration-haemorrhage syndrome |
| K250 | Gastric ulcer, acute with haemorrhage |
| K252 | Gastric ulcer, acute with both haemorrhage and perforation |
| K254 | Gastric ulcer, chronic or unspecified with haemorrhage |
| K256 | Gastric ulcer, chronic or unspecified with both haemorrhage and perforation |
| K260 | Duodenal ulcer, acute with haemorrhage |
| K262 | Duodenal ulcer, acute with both haemorrhage and perforation |
| K264 | Duodenal ulcer, chronic or unspecified with haemorrhage |
| K266 | Duodenal ulcer, chronic or unspecified with both haemorrhage and perforation |
| K270 | Peptic ulcer, site unspecified, acute with haemorrhage |
| K272 | Peptic ulcer, site unspecified, acute with both haemorrhage and perforation |
| K274 | Peptic ulcer, site unspecified, chronic or unspecified with haemorrhage |
| K276 | Peptic ulcer, site unspecified, chronic or unspecified with both haemorrhage and perforation |
| K280 | Gastrojejunal ulcer, acute with haemorrhage |
| K282 | Gastrojejunal ulcer, acute with both haemorrhage and perforation |
| K284 | Gastrojejunal ulcer, chronic or unspecified with haemorrhage |
| K286 | Gastrojejunal ulcer, chronic or unspecified with both haemorrhage and perforation |
| K290 | Acute haemorrhagic gastritis |
| K625 | Haemorrhage of anus and rectum |
| K661 | Haemoperitoneum |
| K762 | Central haemorrhagic necrosis of liver |
| K920 | Haematemesis |
| K921 | Melaena |
| K922 | Gastrointestinal haemorrhage, unspecified |
| M250 | Haemarthrosis |
| N02. | Recurrent and persistent haematuria |
| N837 | Haematoma of broad ligament |
| N920 | Excessive and frequent menstruation with regular cycle |
| N921 | Excessive and frequent menstruation with irregular cycle |
| N922 | Excessive bleeding in the premenopausal period |
| N924 | Excessive bleeding in the premenopausal period |
| N938 | Other specified abnormal uterine and vaginal bleeding |
| N939 | Abnormal uterine and vaginal bleeding, unspecified |
| N950 | Postmenopausal bleeding |
| O67. | Labour and delivery complicated by intrapartum haemorrhage |
| O72. | postpartum haemorrhage |
| R04. | Haemorrhage from respiratory passages |
| R31. | Unspecified haematuria |
| R58. | Haemorrhage, not elsewhere classified |
| S064 | Epidural haemorrhage |
| S065 | Traumatic subdural haemorrhage |
| S066 | Traumatic subarachnoid haemorrhage |

## S1.4 Thromboembolism clinical codes

Below are the ICD-10 codes for thromboembolisms used to identify clinical outcomes in the cohort.

| **ICD-10 code** | **Category or thromboembolism** | **Description** |
| --- | --- | --- |
| G45. | Artherial thromboembolism | Transient cerebral ischaemic attacks and related syndromes |
| G46. |  | Vascular syndromes of brain in cerebrovascular diseases |
| H340 |  | Transient retinal artery occlusion |
| H341 |  | Central retinal artery occlusion |
| H342 |  | Other retinal artery occlusions |
| I513 |  | Intracardial thrombosis |
| I63. |  | Cerebral infarction |
| I64. |  | Stroke, not specified as haemorrhage or infarction |
| I74. |  | Arterial embolism and thrombosis |
| K550 |  | Acute vascular disorder intestine |
| I21. | Myocardial infarction | Acute myocardial infarction |
| I22. |  | Subsequent myocardial infarction |
| G08. | Venous thromboembolism | Intracranial and intraspinal phlebitis and thrombophlebitis (septic (thrombo)phlebitis) |
| H348 |  | Other retinal vascular occlusions |
| I26. |  | Pulmonary embolism |
| I636 |  | Cerebral infarction due to cerebral venous thrombosis, nonpyogenic |
| I676 |  | Nonpyogenic thrombosis of intracranial venous system |
| I801 |  | Phlebitis and thrombophlebitis of femoral vein |
| I802 |  | Phlebitis and thrombophlebitis of other deep vessels of lower extremities (Deep vein thrombosis NOS) |
| I803 |  | Phlebitis and thrombophlebitis of lower extremities, unspecified (Embolism or thrombosis of lower extremity NOS) |
| I808 |  | Phlebitis and thrombophlebitis of other sites |
| I809 |  | Phlebitis and thrombophlebitis of unspecified site |
| I81. |  | Portal vein thrombosis |
| I820 |  | Budd-Chiari |
| I821 |  | Thrombophlebitis migrans |
| I822 |  | Embolism and thrombosis of vena cava |
| I823 |  | Embolism and thrombosis of renal vein |
| I828 |  | Embolism and thrombosis of other specified veins |
| I829 |  | Embolism and thrombosis of unspecified vein |
| K751 |  | Phlebitis of portal vein |
| K765 |  | Hepatic veno-occlusive disease |
| O223 |  | Deep phlebothrombosis in pregnancy (Deep-vein thrombosis, antepartum) |
| O225 |  | Cerebral venous thrombosis in pregnancy |
| O229 |  | Venous complication in pregnancy, unspecified |
| O871 |  | Deep phlebothrombosis in the puerperium (Deep-vein thrombosis, postpartum/Pelvic thrombophlebitis, postpartum) |
| O873 |  | Cerebral venous thrombosis in the puerperium |
| O879 |  | Venous complication in the puerperium, unspecified |
| O882 |  | Obstetric blood-clot embolism (Obstetric (pulmonary) embolism NOS/Puerperal (pulmonary) embolism NOS) |

# S2.0 Cohort

## S2.1 List of SAIL data sources used in this publication

WLGP - Welsh Longitudinal General Practice Dataset

PEDW - Patient Episode Dataset for Wales

WCSU - Welsh Cancer Intelligence and Surveillance Unit

CNIS - Cancer Network Information System

WDSD – Welsh Demographic Service Dataset

ADDE – Annual District Death Extract

## S2.2 Cohort cleaning steps

A consort diagram detailing the cancer patients identified within SAIL and the cleaning steps taken to reach the final cohort


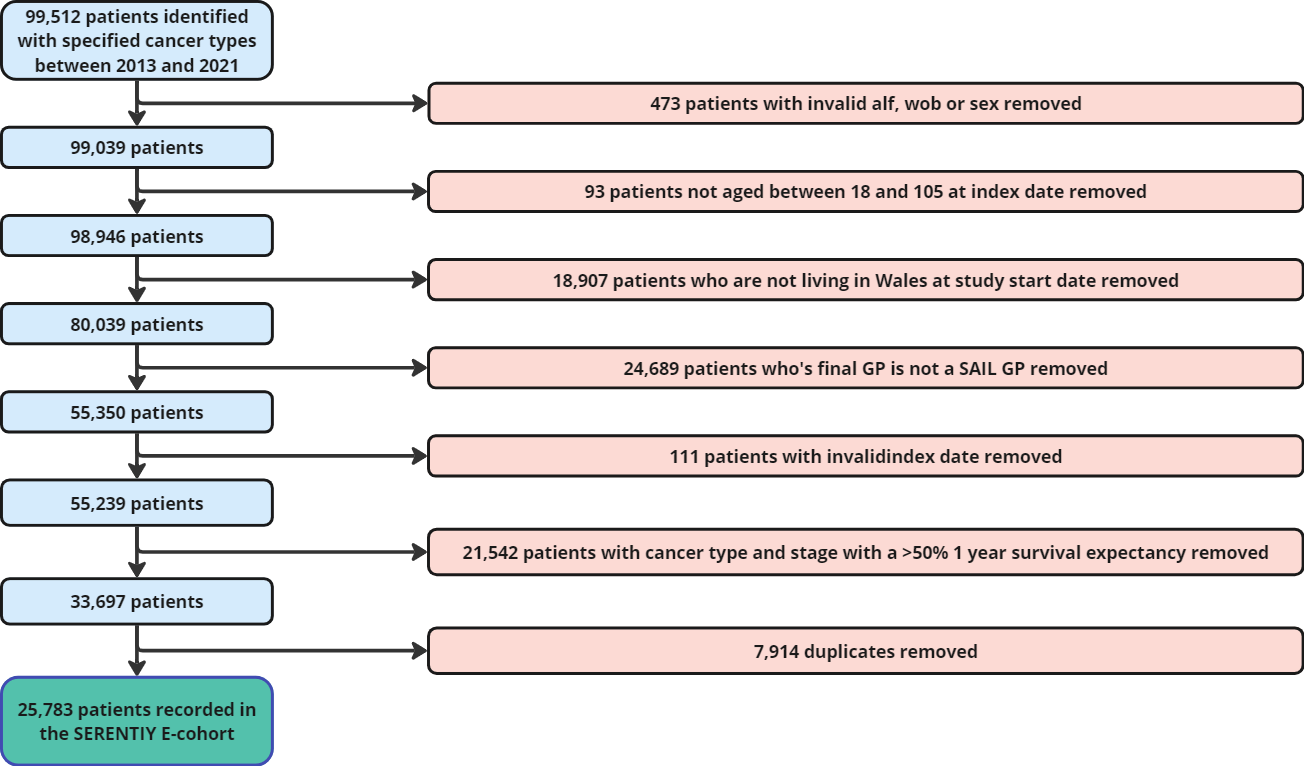


Figure 1 Consort diagram for creation of SERENITY cohort

## S2.3 Prescription interval

| **median** | **mean** | **95%** | **96%** | **97%** | **98%** | **99%** |
| --- | --- | --- | --- | --- | --- | --- |
| 28 days | 28.1 days | 58 days | 63 days | 70 days | 85 days | 116 days |

We identified a population of 30,000 randomly selected individuals within SAIL aged 18-105 with a minimum of 1 ATT prescription. We documented the interval between every prescription event and calculated the mean, median, and 95^th^-99^th^ percentiles.


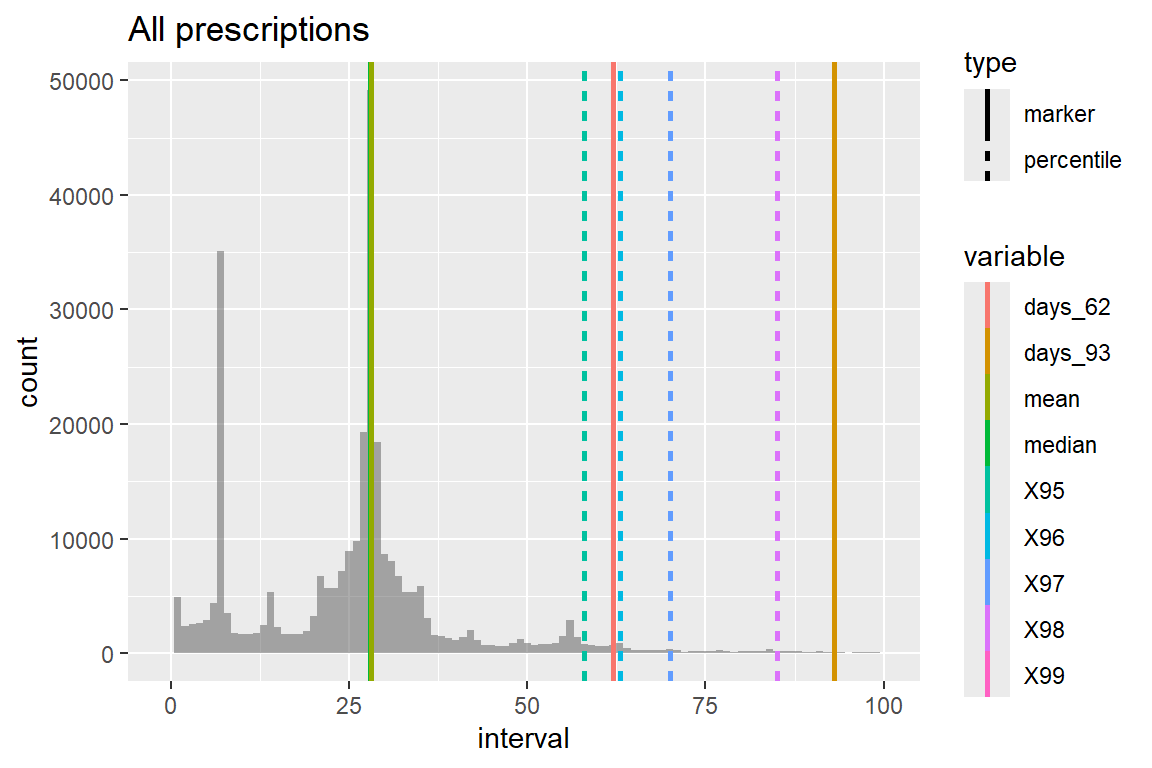


# S3.0 Survival analysis

## S3.1 Survival probability

Here we provide the median survival and Kaplan Meir plots demonstrating the relationship between survival and a range of covariates.

| **Variable** |  | **Median survival (days)** | **n records (n events)** |
| --- | --- | --- | --- |
| **Overall** |  | 145 (142-148) | 25,783 (24,320) |
| **Sex** | Female |  |  |
|  | Male |  |  |
| **ATT usage at index** | ATT exposed at index | 117 (112-122) | 8,260 (7,890) |
|  | ATT unexposed at index | 160 (155-164) | 17,520 (16,440) |
| **Cancer type** | Gastrointestinal – Stage II | 342 (278-431) | 200 (180) |
|  | Gastrointestinal – Stage III | 330 (311-354) | 1,910 (1,670) |
|  | Gastrointestinal – Stage IV | 123 (118-127) | 9,600 (8,970) |
|  | Gynaecological – Stage IV | 244 (204-285) | 660 (630) |
|  | Mesothelial and soft tissue – Stage III | 237 (186-313) | 110 (100) |
|  | Mesothelial and soft tissue – Stage IV | 172 (132-259) | 140 (130) |
|  | Respiratory & thorax – Stage III | 273 (260-285) | 3,240 (3,050) |
|  | Respiratory & thorax – Stage IV | 83 (80-87) | 6,930 (6,770) |
|  | Secondary and unspecified – Stage IV | 59 (51-65) | 910 (890) |
|  | Urogenital – Stage III | 353 (313-424) | 280 (250) |
|  | Urogenital – Stage IV | 165 (147-185) | 1,280 (1,200) |


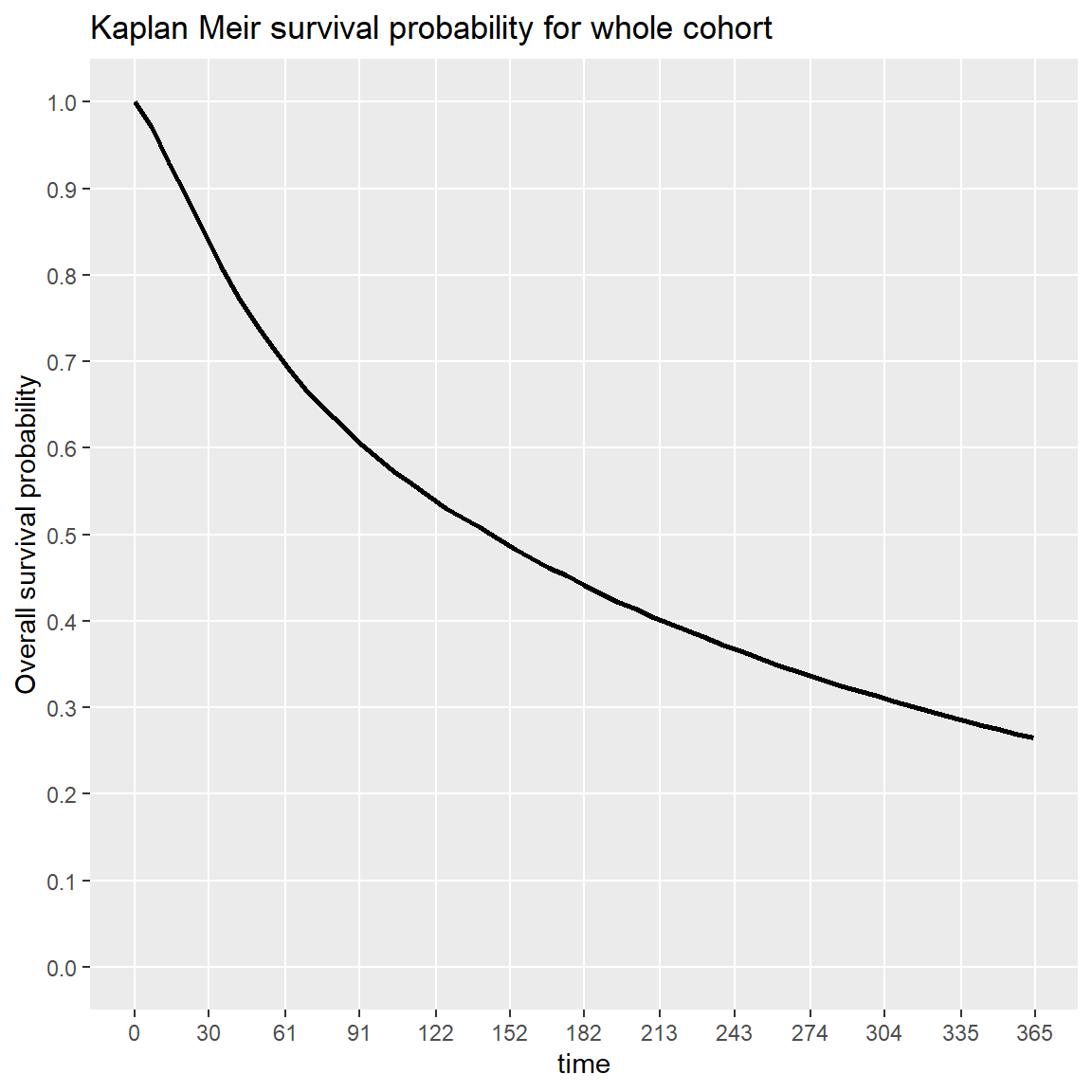


Figure 2 Kaplan Meier survival probability for whole cohort


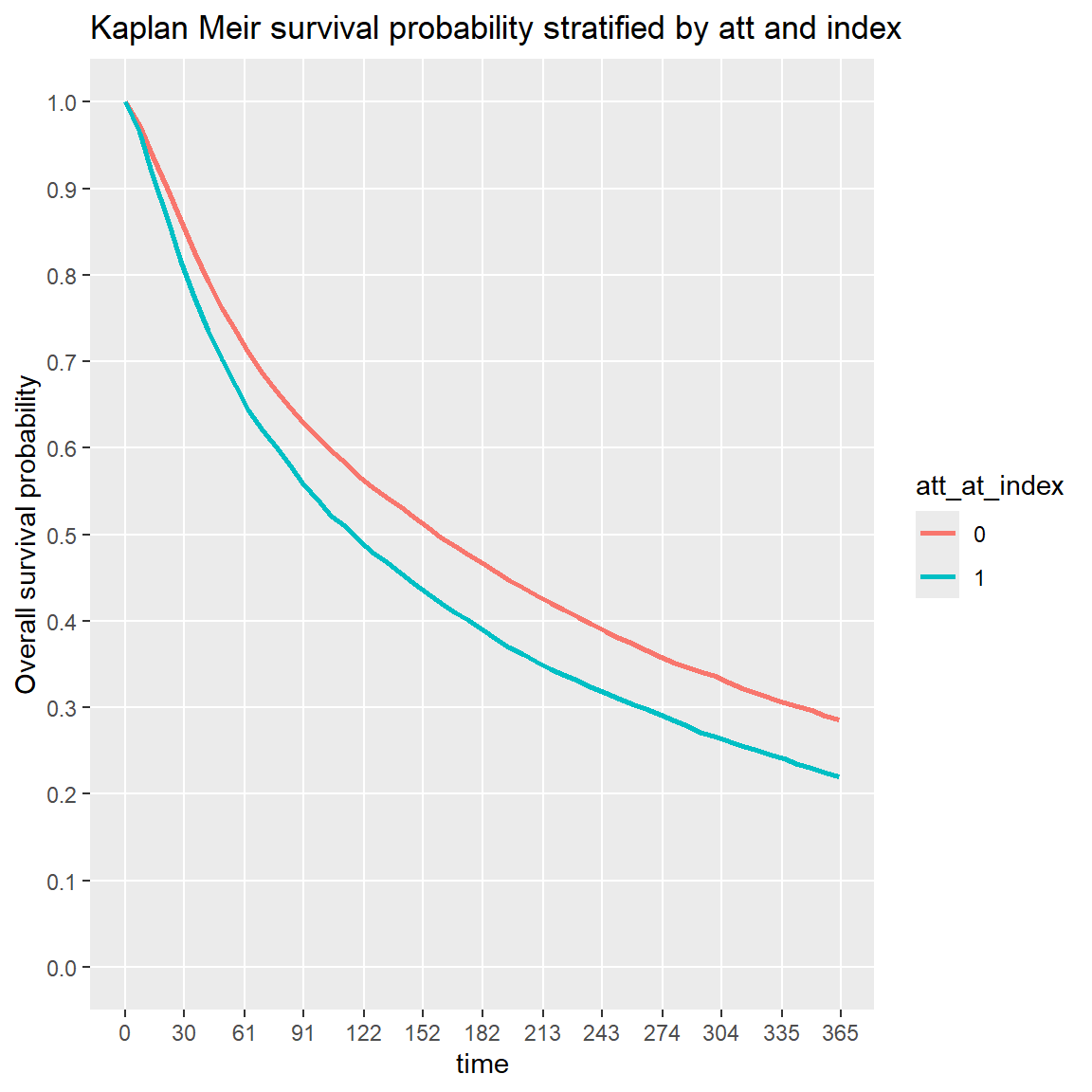


Figure 3 Kaplan Meier survival probability stratified by ATT exposure at index. 0 = unexposed, 1 = exposed.


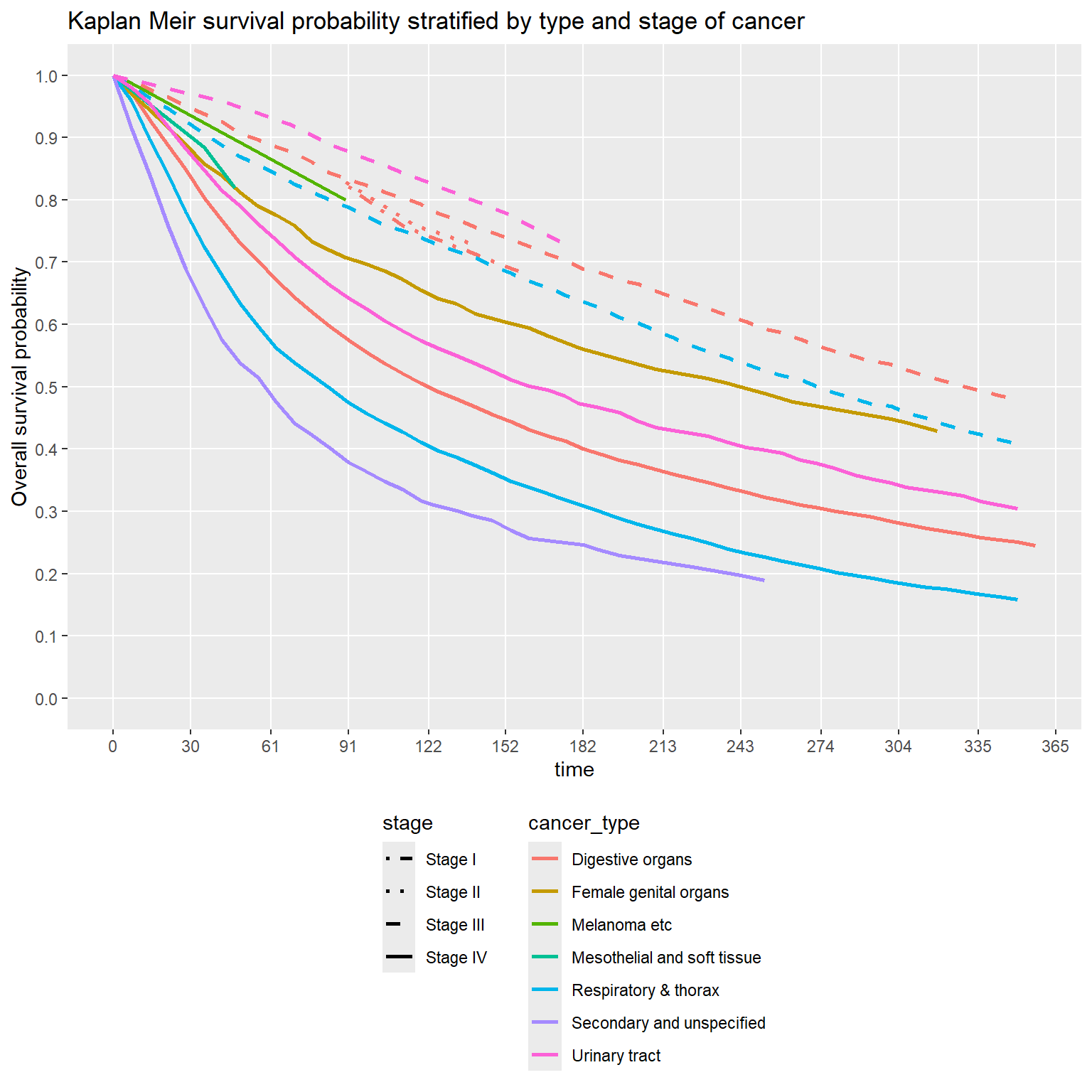


Figure 4 Kaplan Meier curve demonstrating the survival probability of cohort individuals in relation to their cancer and stage.

## S3.2 Cumulative incidence


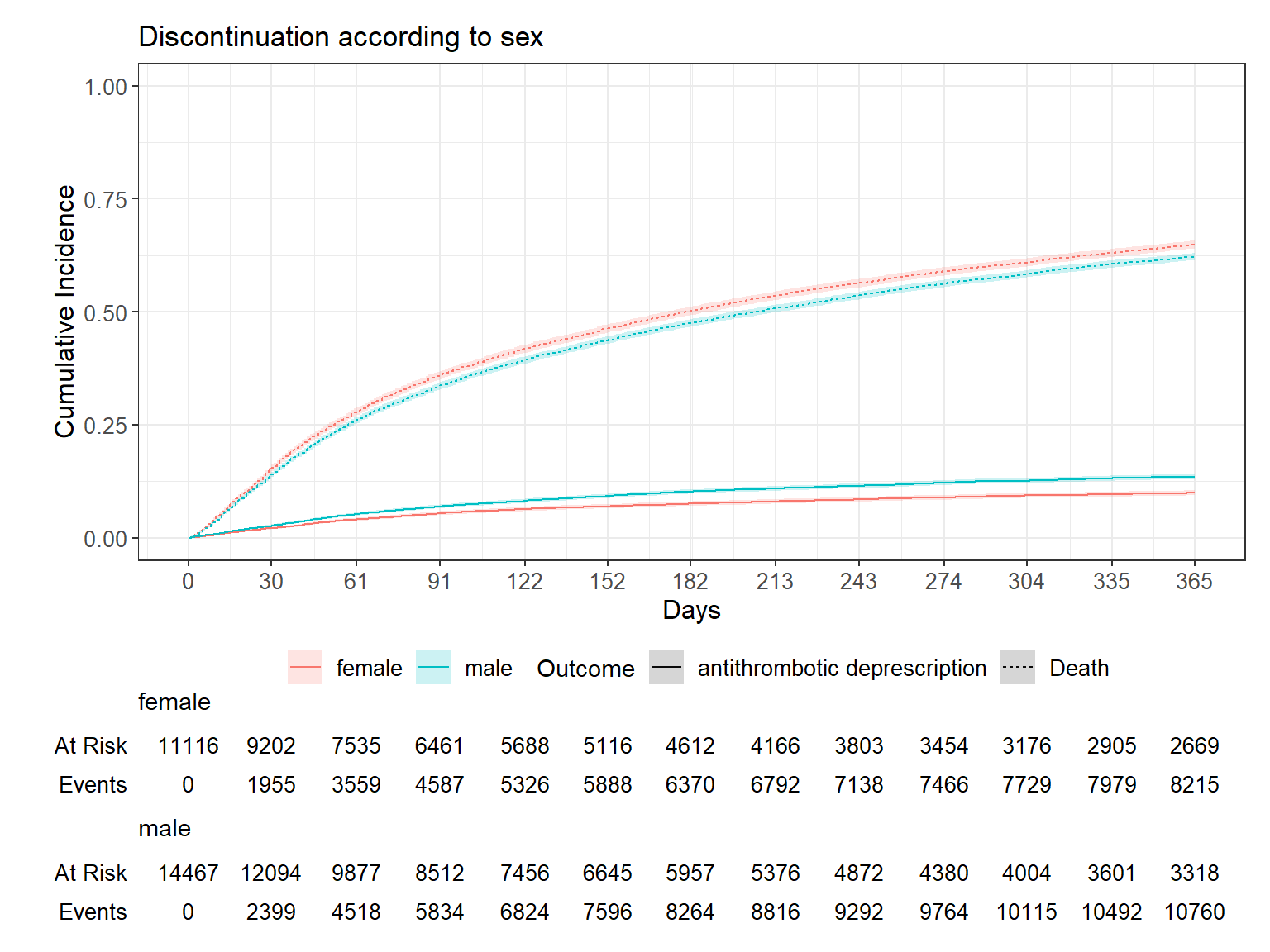


Figure 5 Cumulative incidence of ATT discontinuation stratified by sex and including the competing risk of death


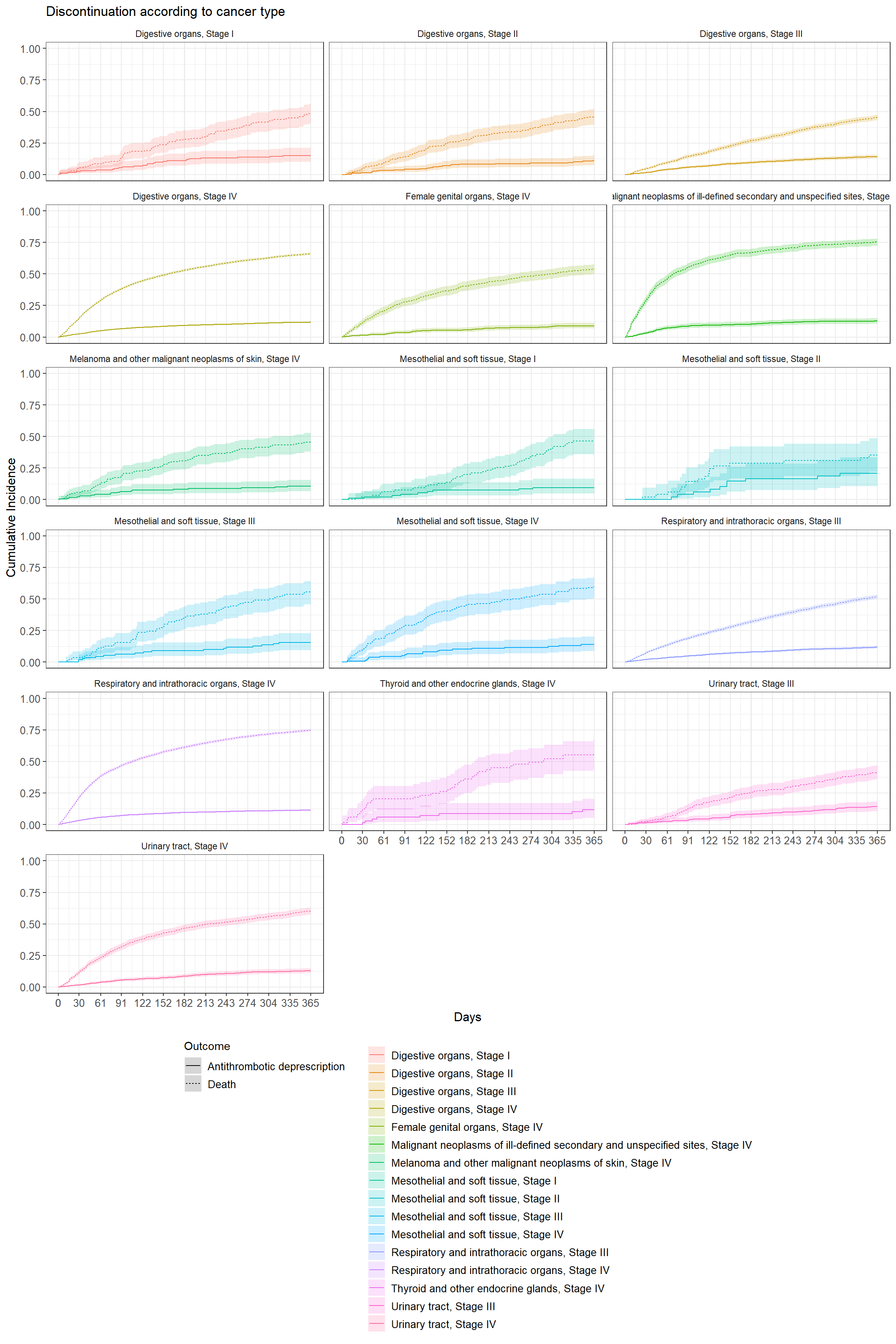


Figure 6 Cumulative incidence of ATT discontinuation stratified by cancer type and stage, including the competing risk of death
